# Patient-reported outcomes and experiences from the perspective of colorectal cancer survivors: meta-synthesis of qualitative studies

**DOI:** 10.1186/s41687-020-00195-9

**Published:** 2020-04-25

**Authors:** Claudia Rutherford, Fabiola Müller, Nasiba Faiz, Madeleine T. King, Kate White

**Affiliations:** 1 The University of Sydney, School of Psychology, Quality of Life Office, Faculty of Science, Sydney, Australia; 2 The University of Sydney, Susan Wakil School of Nursing and Midwifery, Cancer Nursing Research Unit (CNRU), Faculty of Medicine and Health, Sydney, Australia

**Keywords:** Bowel cancer, Systematic review, Patient-reported outcomes, Experiences, Qualitative

## Abstract

**Background:**

Colorectal cancer (CRC) is prevalent in the developed world. Favourable survival rates highlight the need to better understand CRC survivors’ experiences of long-term impacts of treatment, which can in turn inform decision making. This systematic review aimed to identify and synthesise CRC survivors’ experiences of long-term impacts on health-related quality of life.

**Methods:**

We searched Medline, Embase and PsychINFO from inception to January 2019. Qualitative studies describing CRC survivors’ experiences at least 1-year post-treatment were included. Study eligibility, quality assessment (COREQ guidelines), and data synthesis was performed independently by two reviewers and discussed with the study team.

**Results:**

Of 1363 papers retrieved, 20 reporting 15 studies met eligibility. Thematic synthesis produced 12 themes: symptoms, physical, social, psychological and sexual functioning, impact on relationships, informal care needs provided by family/friend, supportive care needs provided by healthcare professional, health care experiences, health behaviour, financial toxicity and occupational experiences. Stoma problems (e.g. leakage, skin irritation) were common in ostomates. Survivors with no/reversed stoma experienced unexpected, long-term altered and unpredictable bowel functioning. Survivors often regulated timing, amount and foods consumed to manage bowel functioning. Less common symptoms included fatigue, impaired sleep and anal pain. Stoma problems and altered bowel functioning impaired survivors’ physical, social, sexual and psychological functioning. Cognitive functioning and heredity issues were not reported in any paper.

**Conclusion:**

CRC survivors experience ongoing symptoms and functioning impairments more than 1-year post-treatment completion. Many survivors find their own ways to manage symptoms rather than seek professional help. Follow-up care for CRC survivors should integrate screening for long-term effects and provide targeted supportive care.

## Introduction

Colorectal cancer (CRC), also known as bowel, colon or rectal cancer, is a leading cause of cancer-related morbidity and mortality worldwide [1]. It is a common malignancy in the developed world, with unhealthy lifestyles and diet contributing to the rising incidence [2]. Screening programs, early detection, and advances in effective treatments have led to a significant increase in CRC survivors, with CRC survivors being one of the most prevalent adult survivor populations [3, 4]. Those diagnosed and treated for early stage disease have a 70–90% 5-year survival rate [3, 4].

Cancer survivorship is an international health policy priority [5], with screening, supportive care needs assessment and patient support services a recognised need [6, 7]. “*An individual is considered a cancer survivor from the time of diagnosis, through the balance of his or her life. Family members, friends, and caregivers are also impacted by the survivorship experience and are therefore included in this definition*” [8]. In 2005, a landmark report from the US Institute of Medicine (IOM) ‘From Cancer Patient to Cancer Survivor: Lost in Transition’ [9] highlighted the need for intervention research to address the serious medical, functional and psychosocial consequences of cancer and its treatments. The report noted that “addressing survivors’ unmet needs and providing clarity around follow up is likely to lead to significant efficiencies in healthcare delivery and potential cost savings.” [9] The IOM report further highlighted the absence of “guidance regarding the functional sequelae that may follow surgical interventions (e.g., colostomy, bowel dysfunction, sexual dysfunction).”

Many individuals who have received treatment for CRC, referred to as ‘CRC survivors’ from hereon, continue to experience physical and/or psychosocial effects of their cancer and its treatments, including physical symptoms [10], functioning impairments (e.g. physical, social, sexual), and fear of cancer recurrence; all of which can negatively impact health-related quality of life (HRQOL) [11, 12]. These effects can become evident during anti-cancer treatment and continue for 1–5 years post-treatment completion (long-term effects), while others may manifest months or even years after completing active treatment (late effects) [13, 14]. Both the number and severity of symptoms contribute to overall symptom burden, and greater symptom burden is associated with a greater reduction in HRQOL [15]. As well as functioning and symptoms, other important aspects of a person’s experience of disease and treatment may directly impact their HRQOL, such as satisfaction with care, unmet needs for information or support services, and psychological adjustment to illness. Often the term HRQOL is used when any *patient-reported outcome* is measured. *“A patient-reported outcome (PRO) is any report of the status of a patient’s health condition that comes directly from the patient, without interpretation of the patient’s response by a clinician or anyone else”* [16]*.* The umbrella term “PRO” tells us the patient is providing the data, but does not tell us what is being measured unless the specific PRO is stated explicitly. Similarly, the umbrella term “HRQOL” does not tell us which aspects of HRQOL are being affected, unless they are stated explicitly.

PROs are important clinical trial endpoints and several PROs have been found to predict survival in CRC survivors above and beyond clinical predictors [17]. Specifically, physical functioning, fatigue, pain and appetite loss predicted overall survival more often than other PROs in metastatic disease (19/27 studies) and emotional well-being and mood predicted overall survival in mixed-stage samples. However, a review of guidelines for CRC survivor follow-up highlighted that most focus on detection of cancer recurrence and assessment of treatment consequences, with little attention placed on identifying and responding to PROs and patient unmet needs [18]. In intervention effectiveness evaluation, HRQOL and other PROs are often not included also. A recent systematic review that included nine studies evaluating palliative pelvic radiotherapy in CRC patients found no reports of PROs or HRQOL [19]. Another review evaluating the effectiveness of chemotherapy +/− bevacizumab in CRC patients did not report HRQOL/PROs [20]. Interestingly, recent recommendations on the treatment of peritoneal metastases of CRC did not include HRQOL/PRO evidence [21] despite the USA Food and Drug Administration (and other) drug and policy bodies requirement for HRQOL/PRO data to support labelling (efficacy) claims [16].

The growing number of CRC survivors, the importance of HRQOL to patients, the potential for PROs to be integral to informing patient-centred CRC treatment decisions and follow-up supportive care, and the critical gaps in evidence about their PROs all point to the importance of understanding the experiences of CRC survivors and their supportive care needs during medium- to long-term survivorship. For the purpose of our review, we considered medium- to long-term survivorship to be at least 12 months post-primary treatment (or > 2 years post-diagnosis of CRC). Our choice to focus on this period was to gain better understanding of lasting disease and treatment effects and supportive care needs. Specifically, we aimed to understand:

1. The medium and long-term impacts (e.g. symptoms and functioning) of treatment for CRC on daily life;
2. The broad positive and negative experiences of CRC survivors at least 12 months post-treatment;
3. The supportive care needs of CRC survivors during post-treatment (≥12 months) survivorship.

We limited our review to qualitative studies as these types of designs allow for in depth enquiry into the experiences of CRC survivors from their perspective. Several reviews synthesising the quantitative evidence about long-term impacts following treatment for CRC have been published [11, 22–26] but a synthesis of the qualitative evidence has not been conducted.

## Methods

Our systematic review of qualitative studies was conducted according to the Preferred Reporting Items for Systematic Reviews and Meta-Analyses (PRISMA) guidance.

### Electronic searches

We searched MEDLINE, EMBASE, and PsychInfo databases from inception to 10 January 2019. Our search strategy comprised a comprehensive set of terms for “quality of life” or “patient-reported outcome,” “bowel cancer,” “experience”, and qualitative methods. Electronic searches were supplemented by searches of the reference lists of the included studies and retrieved review papers. No reviews meeting our eligibility criteria were retrieved.

### Study selection and eligibility criteria

Studies that met the following criteria were included:

- Qualitative study design (e.g. individual interviews or focus groups); mixed method studies (i.e. those that utilised both quantitative and qualitive data collection methods) were considered and the qualitative data was included if it was relevant to our review aims;
- Study underpinned by a clear qualitative framework (e.g. grounded theory);
- Sample was adults with a CRC diagnosis ≥2 years or ≥ 1 year post-primary treatment completion. Studies of mixed tumour samples were included if the CRC sub-group results were reported separately (i.e. CRC survivor experiences explicitly identified, rather than pooled in a synthesis of findings across different tumour groups). Studies of samples with variable time since diagnosis or treatment completion were included if ≥75% of the sample were ≥ 2 years post-diagnosis or ≥ 1 year post-treatment. Similarly, longitudinal studies were included if at least one interview time-point was ≥2 years post-diagnosis or ≥ 1 year post-treatment for ≥75% of the sample and the results were reported separately by time-point;
- Focus of the study was on exploring CRC survivors’ medium to long-term experiences or perceptions of any PRO (e.g. impact of treatment on body image and sexual function) or healthcare service (e.g. management for bowel dysfunction). For studies that reported experiences from the time of diagnosis, only results pertaining to experiences ≥1 year post-primary treatment completion were included.

Studies were excluded if:

- Study participants were receiving palliative care treatment;
- Interviews focused on bowel screening experiences;
- Time since diagnosis or treatment completion was not stated;
- Study participants were ≥ 2 years post-diagnosis or ≥ 1 year post-treatment but the interview asked them to recall and report on their experience at the time of diagnosis or during treatment; and
- It was not possible to discern whether the results reported were patients’ recalled experience of the acute treatment phase or their current ongoing survivorship issues at ≥2 year post-diagnosis or ≥ 1 year post-treatment;

No limits for year, language or geography were applied, however, no non-English published papers met our eligibility criteria. Where papers lacked relevant details, we contacted authors to provide additional information to ascertain study eligibility. If no reply was received or data was not available from the authors, the paper was excluded. For our study, we considered ‘treatment completion’ to be at the end of surgery, chemotherapy or radiotherapy and no palliative treatment received. Some papers reported only time since surgery and did not report whether (some) patients received adjuvant treatment or had a *recurrence* (since their diagnosis). We included these papers if time since surgery was ≥1 year post-treatment.

Retrieved titles and abstracts were screened for eligibility by one reviewer (FM or NF),^1^ and 25% selected at random were cross-checked by a second reviewer (CR). Where abstracts meet eligibility or relevance was ambiguous, papers were obtained and reviewed in full. Full texts were independently reviewed by two reviewers (CR and FM). Disagreements were resolved through team discussion.

### Quality assessment

Included studies were assessment for quality against the 32-item consolidated criteria for reporting qualitative studies (COREQ) checklist [27]. The COREQ checklist assesses reporting of interviews and focus groups in qualitative literature against three main domains: research team and reflexivity, study design, and analysis and findings. Each item of the 32-item checklist is scored 0 = not reported, 1 = partially reported, and 2 = reported, with each paper receiving a total quality score out of 64, which was calculated as a percentage. Thus, higher scores indicate higher reporting quality.

First, two reviewers (FM and NF) independently assessed three papers. They then met with a third reviewer (CR) to compare assessments and discuss disagreements until consensus. Given the high rate of disagreements, the reviewers developed a list of rules for each COREQ item to ensure a standardised rating process, which was adapted iteratively (Online Resource 1). This rule list was applied independently by the reviewers to a second set of papers. The two reviewer ratings were compared and any disagreements resolved. The final agreed standardised rule list was applied to the remaining included papers independently and any disagreements resolved. Disagreement scores ranged between 1 and 10 of the 32 COREQ items per paper, with an average of 5/32 items across the papers.

### Data extraction

A data extraction form was developed, including study aim, sample demographics (e.g. age, gender), tumour characteristics (e.g. stage and type), time since diagnosis or primary treatment completion, interview or focus group topics, and results pertaining to patient-reported outcomes and experiences, including symptoms, functioning (physical, social, psychological, sexual, and cognitive health), impact on relationships, unmet needs, health promotion behaviours (e.g. diet, physical activity), and experience of healthcare. These topics were chosen as important HRQOL components and unmet needs in cancer survivorship [28, 29]. One reviewer extracted data (FM or NF) and a second reviewer (FM, NF or CR) cross-checked extractions against the original paper for accuracy.

### Data synthesis

We used meta-synthesis methods [30, 31] to collate and summarise the evidence across studies. First, each reported qualitative finding was coded to a broad category. A category was determined by grouping common findings (i.e. findings that reflected similar phenomena or variables). For example, the finding of frequent bowel movement and faecal urgency were grouped under the category “bowel symptoms”. Categorising findings was a way of aggregating findings across included studies. Categories that were sufficiently similar in meaning were then generated into synthesised themes. For example, pain and bowel symptoms were grouped under a broad theme called “Symptoms”. Synthesising categories allowed aggregating grouped findings into specific patient-reported themes, providing a summary of the evidence for each theme and generating a framework of the CRC survivor long-term outcomes and experiences. Under each theme, we reported the key findings for stoma, reversed stoma and mixed (e.g. no-stoma and stoma) samples separately. Clinically, there are known different short and long-term bowel function changes for each of these clinical groups. Therefore, it was important to distinguish between them and highlight whether the evidence supported the presence of ongoing symptoms and to identify in which groups they were a problem.

## Results

### Summary of included studies

**Fig. 1:**
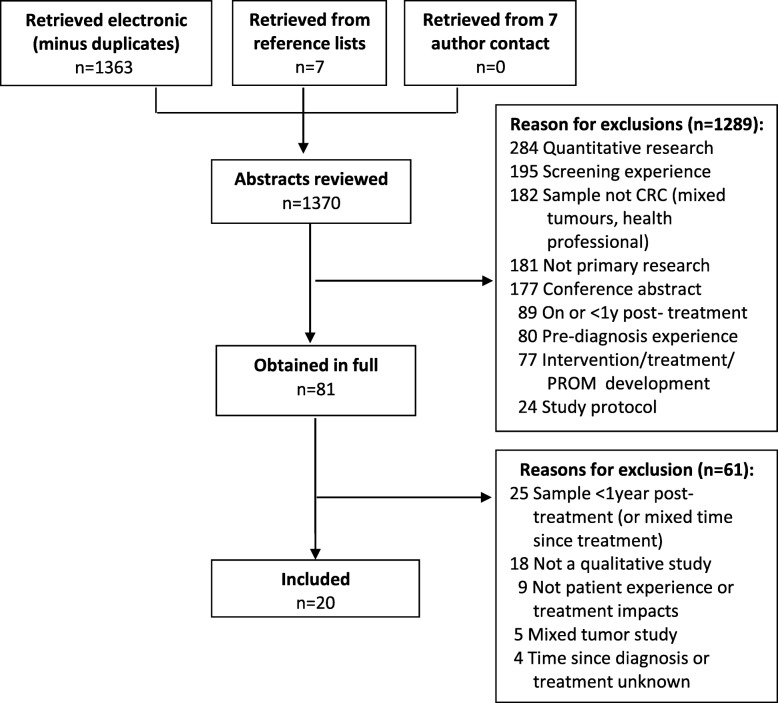
Flow of studies through the selection process

Searches yielded 1370 papers, minus duplicates, of which 81 were considered potentially relevant and 20 met eligibility criteria, reporting on 15 studies (or individual datasets) (Fig. 1); 10 interview only, one focus group, three a combination of interviews and focus groups, and one a combination of field observations and interviews. Where several papers arose from one study, these reported different findings and were grouped together in Table 1. The papers covered surgery alone (*n* = 10), or a combination of treatment modalities including chemotherapy or chemoradiation (*n* = 10). Seven papers included only patients with a permanent ostomy and three papers only patients with a stoma reversal. The 15 studies included participants with colorectal (*n* = 11 studies) or rectal (*n* = 4 studies); none of anal cancer. Participants across studies varied in stage of disease at diagnosis: all papers that reported disease stage were mixed (*n* = 9), ranging from stage I to IV; 11 did not report disease stage. Participants were between 1 and 19 years since diagnosis; four studies included participants between 7 and 26 months post-stoma reversal surgery. Total sample across studies was 328 participants; sample sizes ranged from 5 to 93 participants (approximately 43% male) from health settings across Europe, the United States of America, Canada, Asia, and Australia. Ages ranged from 44 to 85+. Percentage of people aged 50 and older could not be determined from the available data. Only 11 papers reported ethnicity; participants were primarily Caucasian, and one study included only Taiwanese participants.

**Table 1:** Summary of included studies reporting long-term patient-reported outcomes and experiences of colorectal cancer survivors (*n* = 20)

Study	First author (year), country	Study aim	Sample size, tumour type, tumour stage, time since diagnosis or treatment (mean or median and range), mean age and range, % female, ethnicity, eligibility criteria	Treatment	Study design	Study observations
**Studies including patients with an ostomy.**						
**Study 1**	Grant (2011) [32], USA.	Describe how gender shapes the concerns and adaptations of long-term CRC survivors with ostomies.	*n* = 33 CRC with ostomy, stage NR, ≥5y since diagnosis, R 8-19y. M age NR, R 63-76y; *n* = 16 Female, *n* = 29 White Non-Hispanic.	Surgery, specific treatments NR.	Theoretical framework NR, cross-sectional, 8 focus groups, directive/summative content analysis.	Sub-study of a larger quantitative study on HRQOL. Subsample for focus groups was recruited based on gender and high vs. low HRQOL score.
	Sun (2013) [33], USA.	Describe persistent ostomy-specific concerns and adaptations in long-term CRC survivors with ostomies.	*n* = 33 CRC with ostomy, stage NR, ≥ 5y since diagnosis & M since surgery for the 8 focus groups R 8-19y. M age for the 8 focus groups R 63-76y; *n* = 16 Female, *n* = 29 White Non-Hispanic.	Surgery, specific treatments NR.	Theoretical framework NR, cross-sectional, focus groups, content analysis.	Substudy of a larger quantitative study on HRQOL. Subsample for focus groups was recruited based on gender and high vs. low HRQOL score.
**Study 2**	Altschuler (2018) [34], USA.	Discuss how mutuality may affect long-term ostomy caregiving.	*n* = 31 CRC with permanent ostomy, stage NR, ≥ 5y since diagnosis. *n* = 21 ≥ 71y (R 45-85y+), *n* = 17 Female, *n* = 27 Caucasian. Receiving ≥1 h of unpaid caregiving per week because of a health problem or functional impairment.	Surgery, specific treatments NR, *n* = 27 colostomy, *n* = 4 ileostomy.	Theoretical framework NR, cross-sectional, semi-structured interviews, inductive thematic analysis.	Informal caregivers included and interviewed separately from CRC. CRC vs. caregiver data not reported separately in paper.
	McMullen (2011) [35], USA.	Identify factors that hinder or facilitate detection and treatment of ostomy and skin care problems.	*n* = 31 CRC with permanent ostomy, stage NR, ≥ 5y since diagnosis. *n* = 21 ≥ 71y (R 45-85y+), *n* = 17 Female, *n* = 27 White/Caucasian. Receiving ≥1 h of unpaid caregiving per week because of a health problem or functional impairment.	Surgery, specific treatments NR, *n* = 27 colostomy, *n* = 4 ileostomy.	Ethnography, cross-sectional, in-depth interviews (*n* = 31) & repeated field observations (*n* = 6 families), qualitative thematic and matrix analysis.	Informal caregivers interviewed separately from CRC but data from CRC vs caregiver data not reported separately. Interview data and field observation data not reported separately.
**Study 3**	Ramirez (2009) [36], USA.	Shed light on the sexual challenges and adaptations made in the wake of cancer surgery and treatment.	*n* = 30 CRC with permanent ostomy, stage NR, ≥ 5y since diagnosis. Age M 70y (R 44-93y), *n* = 30 Female, *n* = 22 White Non-Hispanic.	Surgery, specific treatments NR.	Anthropological perspective/phenomenology, cross-sectional, semi-structured interviews, analysis methods based on grounded theory.	Sample 100% Female; all heterosexual.
	Ramirez (2014) [37], USA.	Examine how female CRC survivors in the United States articulate their experience living with an ostomy as an erosion of full adult personhood.	*n* = 30 CRC with permanent ostomy, stage NR, ≥ 5y since diagnosis. Age M 70y (R 44-93y), *n* = 30 Female, *n* = 22 White Non-Hispanic.	Surgery, specific treatments NR.	Theoretical framework NR, cross-sectional, semi-structured interviews, analysis methods NR.	Sample 100% Female; all heterosexual.
**Study 4**	Altschuler (2009) [38], USA.	Understand how a range of aspects of intimacy and sexuality is affected by having an ostomy as a result of CRC.	*n* = 22 CRC with permanent ostomy, stage NR, ≥ 5y since diagnosis. Age M 70y (R 44-93y)^a^, *n* = 22 Female, *n* = 22 White Non-Hispanic ^a^. Married or partnered.	Surgery, specific treatments NR.	Theoretical framework NR, cross-sectional, semi-structured interviews, analysis methods NR.	Sample 100% Female and married/partnered; all heterosexual. Subsample of Ramirez, 2009/2014.
**Studies including patients with a reversed ostomy.**						
**Study 5**	Desnoo (2006) [39], UK.	Explore the physical and psychosocial issues of patients with anterior resection syndrome, strategies patients used when adapting to the chronic problems of the syndrome.	*n* = 7 RC with reversed ostomy, stage NR, M 11 m (R 7-12 m) since reversal surgery. Age M 70.7y (R 65-78y), *n* = 5 Female, race NR. No metastases, no local recurrence.	*n* = 7 anterior resection with temporary ileostomy, *n* = 5 adj C(R)T.	Grounded theory, cross-sectional, semi-structured interviews, constant comparative method.	
**Study 6**	Owen (2008) [40], UK.	Investigate how the experiences of having a stoma and subsequent stoma reversal affect the lives of participants.	*n* = 5 CRC with reversed ostomy, Duke Stage A or B at diagnosis, M 11 m (R 8-13 m) since reversal surgery. Age M 67.4y (R 60-78y), *n* = 3 Female, *n* = 5 Caucasian. No CT.	Surgery (specific treatments NR).	Phenomenology/Grounded theory, cross-sectional, semi-structured interviews, interpretative phenomenological analysis/thematic analysis.	
**Study 7**	Reinwalds (2018) [41], Sweden.	Illuminate what it means to live with a resected rectum due to RC, after reversal of a temporary loop ileostomy.	*n* = 10 RC with reversed ostomy, stage NR, M 15.2 m (R 12-20 m) since reversal surgery. Age M 71.6y (R 56-84y), *n* = 6 Female, race NR. No postop complications, no recurrent disease.	*n* = 10 anterior resection with loop ileostomy, *n* = 5 nadj RT of which *n* = 4 adj CT.	Phenomenological hermeneutical, cross-sectional, in-depth interviews, phenomenological hermeneutical method/ thematic structural analysis.	
**Studies including patients with and without a (reversed) stoma.**						
**Study 8**	Hardcastle (2018) [42], Australia.	Explore CRC survivors’ information and support needs in relation to health concerns and health behaviour change.	*n* = 24 CRC, n = 6 ostomy, *n* = 6 Stage A, *n* = 8 Stage B, *n* = 7 Stage C, *n* = 3 missing, M 25.25 m (SD 9.96) since diagnosis & ≤ 2y since tx completion. Age M 69.38y (R 63-77y), *n* = 13 Female, race NR. Increased risk for cardiovascular disease ^f^.	Surgery NR, *n* = 10 adj CT, *n* = 3 adj RT, n = 3 adj CT/RT.	Theoretical framework NR, cross-sectional, semi-structured interviews, inductive thematic analysis/ content analysis.	
	Hardcastle (2017) [43], Australia.	Explore CRC survivors’ health perceptions following cessation of active treatment and explore factors influencing participation in health-promoting behaviours that may help reduce cardiovascular disease risk.	*n* = 24 CRC, *n* = 6 ostomy, *n* = 6 Stage A, *n* = 8 Stage B, *n* = 7 Stage C, *n* = 3 missing, M 25.25 m (SD 9.96) since diagnosis & ≤ 2y since tx completion. Age M 69.38y (R 63-77y), *n* = 13 Female, race NR. Increased risk for cardiovascular disease ^f^.	Surgery NR, *n* = 10 adj CT, *n* = 3 adj RT, *n* = 3 adj CT/RT.	Theoretical framework NR, cross-sectional, semi-structured interviews, inductive thematic analysis.	
	Maxwell-Smith (2017) [44], Australia.	Explore CRC survivors’ experiences and barriers towards physical activity among those with comorbidities, as a precursor to developing effective patient-centered interventions.	*n* = 24 CRC, *n* = 6 ostomy, *n* = 6 Stage A, *n* = 8 Stage B, *n* = 7 Stage C, *n* = 3 missing, M 25.25 m (SD 9.96) since diagnosis & ≤ 2y since tx completion. Age M 69.38y (R 63-77y), *n* = 13 Female, race NR. Increased risk for cardiovascular disease^f^.	Surgery NR, *n* = 10 adj CT, *n* = 3 adj RT, n = 3 adj CT/RT.	Theoretical framework NR, cross-sectional, semi-structured interviews, inductive thematic analysis.	
**Study 9**	Ball (2013) [45], USA.	Understand men’s perceptions of how RC treatment impacts their sexual functioning and how men manage sexual dysfunction.	*n* = 13 RC, *n* = 7 reversed ostomy, 6.5% stage I, 29% stage II, 64.5% stage III^b,c^, MD 6.4y since tx. Age MD 67y (R 47-82y), *n* = 13 Male, n = 13 Caucasian. No disease or recurrence.	n = 13 surgery, n = 7 CT, n = 5 RT.	Theoretical framework NR, cross-sectional, semi-structured interviews (n = 6) & focus groups (n = 7), thematic analysis.	100% Male sample.
**Study 10**	Lu (2017) [46], Taiwan.	Explore the lived experiences of post-operative RC patients with altered bowel function.	n = 16 RC, n = 11 reversed ostomy, stage^b^ n = 1 Tis, n = 3 T0, n = 3 T1, *n* = 4 T2, n = 5 T3, R 1w-36 m since surgery. Age M 55y (R 40-75y), *n* = 8 Female, *n* = 16 Taiwanese^b^. ≥1 postop altered bowel function symptom.	n = 16 low anterior resection^b^, *n* = 9 nadj CRT^b^, n = 1 nadj RT^b^, n = 8 adj CT^b^.	Husserlian descriptive phenomenological approach, cross-sectional, semi-structured interviews, thematic analysis using Colaizzi’s seven-step method.	Relatively young sample; Asian sample.
**Study 11**	McGeechan (2018) [47], England.	Explore the psychosocial and physical consequences of living with CRC as a chronic illness and how this changes survivor’s views and plans for their future, over time.	n = 6(T1)/n = 5(T2) CRC, n = 1 permanent ostomy, stage n = 1 T2^d^, n = 1 T3N1^d^, n = 4 unknown^d^, < 1-4y since diagnosis^d^. Age M 59.8y (44-72y)^d^, *n* = 2 Female^d^, n = 6 White-British^d^.	n = 6 surgery, n = 1 CT^d^.	Theoretical framework NR, longitudinal (2 post-tx assessments over 6 m), semi-structured interviews, interpretative phenomenological analysis.	Data extraction based on second interview (T2), which includes the patient with permanent ostomy.
**Study 12**	Drott (2016) [48], Sweden.	Explore CRC patients’ experiences of oxaliplatin-induced neurotoxic side effects and how these side effects influence their daily lives over time.	n = 10(T1) CRC (n = 9 CC, n = 1 RC), at least n = 1 ostomy^b^, stage II-III, T4 12m since CT. Age M 61y (44-68y), *n* = 7 Female, n = 10 Swedish^b^. For n = 4 at T4^b^: n = 4 CC, n = 1 ostomy, n = 4 stage III, 12 m since CT. Age M 64y (R 61-67y), n = 2 Female, n = 2 Swedish. No neurotoxic side-effects, nadj or palliative tx, or metastasis.	n = 10 surgery with adj CT (Folfox or Xelofox).	Theoretical framework NR, longitudinal (4 post-tx assessments over 12 m), semi-structured interviews, thematic analysis.	Data extraction based on final interview (T4).
**Study 13**	Sun (2015) [49], USA.	Explore specific strategies used by CRC survivors to manage bowel dysfunction.	*n* = 92 CRC with permanent ostomy or anastomosis, stage NR, ≥ 5y since diagnosis. Age Interview subsample M 70y (R 44-93y)^e^, age Focus group subsample NR, gender Interview subsample *n* = 30 Female, gender Focus group subsample NR, race Interview subsample *n* = 22 White Non-Hispanic ^e^, race Focus group subsample NR.	Surgery, specific treatments NR.	Theoretical framework NR. Mixed method study: cross-sectional, focus groups (*n* = 62) & interviews (n = 30), content analysis.	Subsample for focus groups was recruited based on high vs. low HRQOL score. Data from n = 62 focus group patients likely overlaps with Study 1 (Grant, [32]; Sun, [33]). Data from n = 30 interviews is likely the same data as Study 3 (Ramirez, [36, 37]).
**Study 14**	Urquhart (2012) [50], Canada.	Explore the views of breast and CRC survivors on their routine follow-up care, with respect to needs, preferences, and quality of follow-up, and their views on cancer specialist– compared with family physician–led follow-up care.	Descriptives for CRC subsample: n = 10 CRC, n = 4 permanent ostomy & n = 4 (to be) reversed ostomy, stage NR, 12–72 m since diagnosis & ≥ 3 m since tx. Age NR, n = 4 Female, race NR. Receiving routine follow-up care. No current disease, no complications from primary tx.	Treatment NR.	Phenomenology, cross-sectional, semi-structured interviews (n = 4 CRC) & focus groups (n = 6 CRC), thematic analysis.	Data extraction based on results presented for CRC.
**Study 15**	Burden (2016) [51], NR.	Explore individuals’ relationships with food along with their views and experiences of nutritional issues throughout the treatment and disease continuum for CRC.	*n* = 25 CRC, n = 8 ostomy, stage n = 1 Tubolovillous, n = 1 TNM stage 1, n = 2 stage 2, *n* = 14 stage 3, n = 7 stage 4, MD 17 m (R 7-30 m) since surgery. Age M 67.7y (SD 12.4), n = 7 Female, race NR.	n = 25 surgery, n = 9 adj CT, n = 9 nadj RT, n = 3 missing.	Phenomenology, cross-sectional, semi-structured interviews, thematic analysis.	Data extraction based on post-tx experiences.

*CC* Colon cancer, *CRC* Colorectal cancer, *RC* Rectal cancer, *Tx* Treatment, *CT* Chemotherapy, *RT* Radiotherapy, *CRT* Chemoradiotherapy, *adj* adjuvant, *nadj* neoadjuvant, *NR* Not reported, *R* Range, *M* Mean, *MD* Median, *SD* Standard deviation, *w* week, *m* month, *y* year(s), *n* sample size

^a^Descriptives based on full sample (*n* = 30). NR for subsample (*n* = 22)

^b^Additional data provided by author(s)

^c^Percentage based on valid/non-missing values

^d^Descriptives based on full sample (*n* = 6). NR for subsample (*n* = 5)

^e^Based on Ramirez [36, 37]

^f^American Society of Anesthesiologists physical status score = 2 or 3

### Methodological study quality

**Fig. 2:**
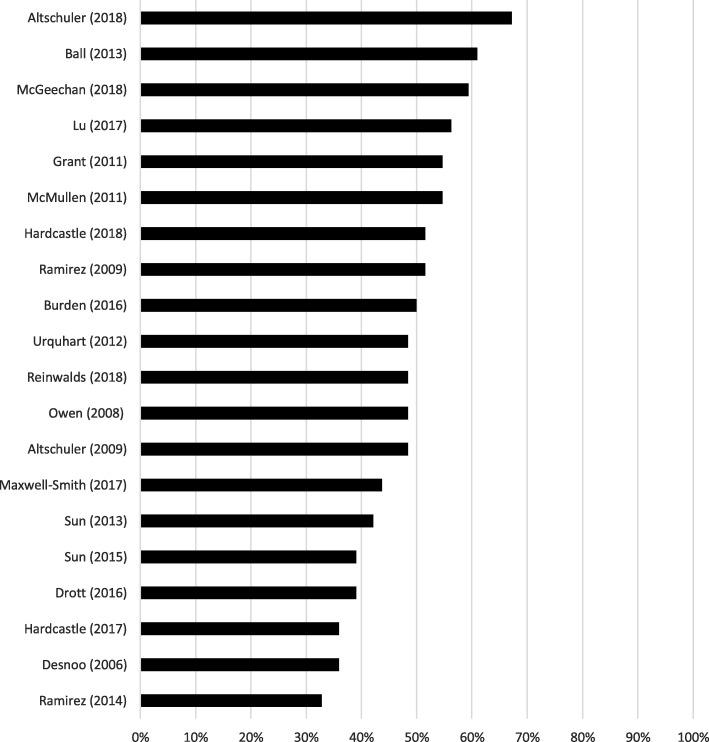
Quality score per included paper (*n*=20)

**Fig. 3:**
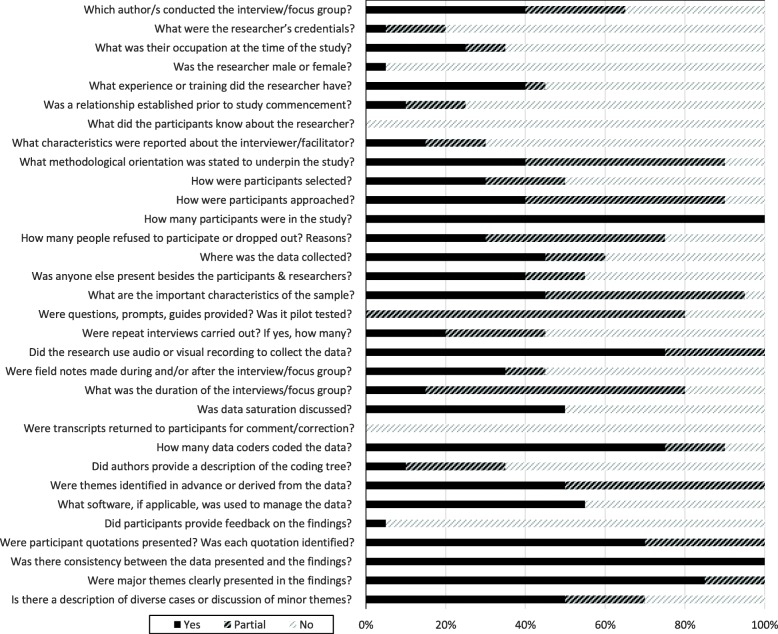
Quality rating across included papers (*n*=20) per COREQ item

Quality scores ranged from 33% [37] to 67% [34] (Fig. 2). No papers met all quality criteria, however, some quality items were adequately addressed by all studies; notably, all studies reported the number of study participants and there was consistency between the data presented and study findings reported (Fig. 3). No studies provided information about what the study participants knew about the researcher or whether participants commented on and made any corrections to the transcripts of their interviews. Further reporting limitations arose from inadequate reporting of the gender, credentials and occupation of the researchers; whether a relationship was established between the interviewer and study participants prior to study commencement; lack of characteristics about the interviewer/facilitator; description of the coding tree; and whether participants provided feedback on the study findings (Fig. 3).

### Conceptual framework of PROs important in CRC survivorship

**Fig. 4:**
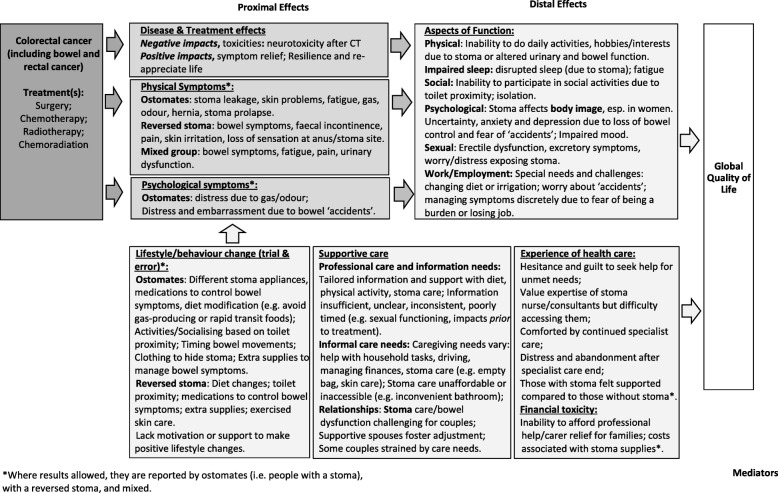
Conceptual framework of patient reported outcomes important in colorectal cancer survivorship

We synthesise and present the results across studies according to 12 themes. All themes and their descriptive components are described below from the perspective of CRC survivors. These 12 themes informed our conceptual framework of PROs important in CRC survivorship (Fig. 4).

#### Physical symptoms

Of the 20 included papers, 19 reported symptoms but only eight (explicitly) investigated CRC survivors’ physical symptoms [32–35, 37, 39–41] including one specifically focused on patients with a permanent [35] and three on patients with a reversed stoma [39–41]. Survivors with a permanent stoma most commonly reported leakage from the stoma bag (e.g. due to equipment failure, diarrhoea) or skin issues (e.g. skin breakdown at stoma site, poor placement of skin barrier) [32–35, 37]. Indeed, one participant described their faecal incontinence as: “Ahhh! I stood at the counter in the store and I just felt help, no! It just came! And I shoved my things away and headed out. Then it was diarrhea! It ran down my legs and I was wearing pale pants. And I met a woman and she looked and I thought, yes, well, let her look I cannot help it …Such stuff is so embarrassing!” [41] Other stoma-related problems included sleep disruption (e.g. due to leakage or adjusting sleeping position to accommodate full stoma bag), fatigue, and (distressing) gas and odour [32, 33, 35, 37]. Patient-reported long-term (i.e. 5 years) complications of stoma included hernias (requiring surgical repair) and stoma prolapse [33].

Survivors with a reversed stoma most commonly reported bowel symptoms, such as frequent, irregular and nightly bowel movements, loss of control over bowel function (e.g. inability to hold bowel movements for more than a few minutes or to distinguish flatus from solids), faecal incontinence (from straining to large volumes) that ranged from occasional to more frequent, and altered stool texture [39–41]. Survivors’ altered bowel function caused pain from skin irritation and excoriation around the anus, pain and numbness in legs from prolonged bowel movements, fatigue, and disrupted sleep [41].

The five papers of mixed stoma/non-stoma survivors reported bowel symptoms, including diarrhoea, constipation, excess gas, irregular bowel patterns, frequent defecation, faecal urgency, and faecal incontinence [42, 45, 46, 49, 50] that were more common in people without a stoma compared to those with a stoma [43]. Other commonly reported symptoms included fatigue [44, 45, 47, 48, 50], (anal) pain [45–47, 50], loss of anal awareness, altered urinary function (i.e. urinary urgency, leakage, poor bladder control, hesitant urination), impaired sleep from altered bowel and urinary function [46], and prolonged weight loss after surgery [51]. One study found neurotoxicity persisted > 1 year after oxaliplatin-completion, with survivors reporting distressing sensations in hands and feet that ‘sapped’ their energy [48].

#### Physical functioning

Nine papers reported problems with physical functioning [32, 37, 39, 41, 42, 45, 46] lu [47, 51] of which five aimed to explore physical functioning issues [32, 39, 42, 46, 47]. Two studies of rectal cancer survivors with a stoma reversal reported disrupted sleep and frequent toilet visits for reassurance due to difficulty distinguishing between flatus and solid stools [39, 41]. Shortly after stoma reversal, many survivors regretted the reversal. A year on, three of 10 considered re-establishing their stoma because of the challenged of altered bowel function. However, one male did not want the stoma back due to stoma leakage but associated the stoma with greater control and freedom [41]. Some reported the stoma affected physical activity and caused them to stop various activities such as swimming [32]. Others were able to resume activities ‘after having adjusted to the ostomy’ (e.g. hiking, gardening, bowling).

The seven papers of mixed stoma/non-stoma survivors most commonly reported frequent, urgent or unexpected bowel movements [33, 34, 37, 42, 46, 47, 51]. Altered urinary and bowel function impaired the ability to take part in everyday activities (i.e. work and travel), social activities and sleep, and disrupted mood, caused distress and uncertainty, and reduced HRQOL [34, 42, 46], mainly due to needing to be close to a toilet [42]. Although common problems generally, weight loss, fatigue [47, 51], pain or the ostomy [47] were less commonly attributed to inability to participate in everyday activities.

#### Social functioning

Thirteen papers reported problems with social functioning [32, 34, 37, 39–43, 45–47, 49, 51] of which five [32, 37, 40, 41, 46] aimed to explore social functioning issues. All four studies of rectal cancer survivors reported social functioning issues [39, 41, 45, 46]. Three papers found survivors with a stoma avoided social situations (e.g. stayed home [34, 39]) for fear of embarrassment from a leaking stoma bag, or disrupting others when using the toilet at night [40]. Frequent toileting and the need to be in close proximity of a toilet were the main reasons for social avoidance [32, 39, 40] and impacted on how survivors communicated their symptoms or disclosed their ostomy to others (e.g. use of euphemisms to avoid embarrassment) [39]. Survivors with a stoma often developed strategies to overcome the negative social impact to enable social participation [32, 37, 39]. Strategies included a positive attitude, timing toileting relative to social activities, and adjusting clothing to not reveal their stoma [32]. Others chose to explain their toilet frequency and bowel symptoms to their friends to help them understand their new habits, which enabled socialising [41]. Survivors used “trial and error” for controlling bowel symptoms, and tried different ostomy appliances, irrigation to control timing of emptying, clothing adjustments and changing food consumption habits [37]. These strategies helped with being able to work, travel and socialise, however they were not universal solutions for all survivors. Travel was particularly challenging, causing distress and uncertainty [45], and the need for packing extra supplies.

Four papers of mixed stoma/non-stoma survivors commonly reported needing to be close to a toilet [42, 43, 46], and consequently, avoided socialising and holidays due to having to manage odour, suboptimal bathroom facilities, and dietary intake [51], or planned social activities around toilet proximity [46]. Avoidance was commonly reported, which caused many to “miss out” on social activities as “…I’ve cut myself off from something that might be an enjoyable activity” [49]. Other strategies to enable social participation included changes in eating times, portion sizes, use of anti-diarrhoeal medications to reduce faecal output [51], or planning irrigation time [47]. One survivor described the lengths she went to maintain her social life however futile: “I take knickers in the car…a decent toilet roll…it’s an awful way to live and there’s no stopping it…I don’t see friends anymore…they just stopped asking to go out for lunch” [43].

#### Psychological functioning

Seven papers reported psychological issues [32–38]. Survivors with a permanent stoma experienced ongoing distress, anxiety, depression, embarrassment, and uncertainty due to potential bowel ‘accidents’, odour and noise from the stoma in public [32–35, 37]. Predominantly for women, having a stoma affected their body imagine [32, 36] but emotional and psychosocial support from their partners facilitated adjustment to their stoma [38]. Humour, resilience, and learning to care for the stoma also facilitated adjustment [32, 37].

Patients with a reversed stoma reported persistent distress because of their impaired bowel functioning [39–41]. This was partly due to expecting to return to normal function after a reversal but instead experienced ongoing bowel symptoms [39–41]. Some survivors adapted well, accepting their “new normal” [40] and described their ongoing bowel problems as a “small price to pay” for being alive [39] or applied coping strategies such as humour [41]. “Erratic” bowel patterns caused frustration, loss of control, fear of ‘accidents’, and mood changes. Bowel symptoms in public were embarrassing [39, 41], and survivors felt relief when incontinence occurred in the privacy of their own home [41]. Ongoing bowel symptoms caused worry about cancer recurrence [39, 41], which continued despite reassurance of no recurrence during routine follow-up. Bowel symptoms were a “constant reminder of cancer” [41] yet all survivors in one study preferred their ongoing bowel symptoms to living with an ileostomy [39].

Six papers of mixed stoma/non-stoma survivors also reported distress from ongoing bowel symptoms, including anxiety, uncertainty, being confined to the toilet [43, 45, 46], fear and embarrassment due to bowel and urinary “accidents” [46, 49], and fear of cancer recurrence [43, 47]. In one study, weight loss caused despair, shock, and a changed “perception of self” [51]. Others adjusted through faith, positive thinking, or reprioritising their life (e.g. spend more time with family) [46, 47].

#### Sexual functioning

Six studies reported problems with sexual functioning [32, 36, 37, 45, 46, 50], of which four were of survivors with a stoma [32, 36, 37, 50]. Survivors with a permanent stoma reported problems with sexuality, intimacy and not having an active sex life. This was attributed to complications of treatment (e.g. being “burnt up” from radiation [32]) or self-imposed abstinence from intimacy or sexual activity. For example, one women felt reluctant to undress in front of a man because of a previous partner’s reaction upon seeing the stoma [32]. Another expressed a strong ‘aversion’ to her stoma, reporting using feminine hygiene spray to “blast that sucker” and stated wanting to be cremated with it when she died so that the stoma was “burned up and gone!” [37]. Older women in existing relationships felt supported by their partners and accepted their ‘changed body’ [37]. However, some single women felt uncomfortable starting a new sexual relationship and worried about a potential new partners’ reaction to the stoma [37]. Having to reveal the stoma to a new partner was compared with revealing a missing limb or having AIDS [37].

In one study [36], women with no long-term sexual difficulties (*n* = 11/30) reported their stomas did not interfere with sexual participation. These women experienced difficulties with sexual functioning shortly after surgery but resumed intercourse with “few minor modifications” such as using a new bag or emptying bag before intercourse so that “…he’s not feeling faeces…that would make me feel gross” [36]. Others checked the bag was in place before intercourse for fear of accidents/leakage or covered the bag to keep it stable (‘accident’ prevention) and hidden from view. Several women attributed their resumed sexual activity to partners’ support and acceptance of their “reconfigured body”. Women with long-term sexual difficulties (*n* = 7/30) found intercourse impossible or painful due to vaginal changes following cancer treatment. Their sexual difficulties were distressing and painful, and they felt undesirable because of the stoma bag. Some women refrained from sexual activity post-surgery (*n* = 9/30), believing the stoma made it difficult to have a sexual partner (i.e. smell making one undesirable, poor adjustment to stoma by new partner). For women that described their impaired sexual functioning as less problematic, resuming intercourse was less central to their relationship as they were grateful to be alive. Other couples adjusted their sex life (i.e. masturbating, oral sex instead of intercourse), which was an acceptable alternative for some but not all. For example, for one women for whom sex was an important part of her relationship found different positions, lubricants and a vaginal dilator post-surgery did not help with her sexual difficulties and was extremely painful. This women had wished that she had been given more information about the potential long-term impact of surgery and radiation on her sexual functioning. Others described intercourse as “no longer important” for a harmonious relationship and attributed their lack of interest in intercourse to aging [36].

Two studies explored sexual function in rectal cancer survivors. One study of 13 older (67 median years) men with rectal cancer attributed sexual dysfunction to increasing age rather than their CRC, which facilitated coping (e.g. readjusted expectations about sexual functioning) [45]. In another study, loss of intimacy was attributed to erectile dysfunction (e.g. no ‘hard and long-lasting erection’ sufficient for intercourse) and excretory symptoms [46].

#### Impact of stoma or bowel problems on relationships

Survivors with stomas frequently discussed the value of a supportive partner, both for practical stoma care and emotional adjustment to having a stoma (e.g. partners’ acceptance of “altered body” [32–35, 37, 38]). However, a stoma was challenging for some couples, contributing to relationship breakdown due to high caregiver burden [38] or affected sexual relationships [33, 36, 38]. While a survivors’ partner seemed the most likely source of positive support, not all partners were supportive (e.g. finding the wounds disgusting, feeling depressed by high care demands) or contributed to stoma care [35, 38].

Survivors with a reversed stoma reported that family and friends were important sources of support [47, 50]. However, survivors’ altered bowel functioning impacted their relationships, contributing to tension between couples (e.g. projecting frustration onto the significant other [41]), practical implications such as partners needing to take over household chores, or frequent toilet visits throughout the night disrupting family members’ sleep [46].

#### Informal care needs (family support)

In one study, half of the survivors received help with stoma care [34]. However, some received caregiving without a high need for support, while others needed more support than they currently received [34]. Caregiving tasks included emptying the faecal contents from the stoma bag, strategies to decrease skin irritation (e.g. cleaning the peristomal skin), seeking assistance from nurses, help with household tasks, and driving or managing finances [34, 35]. Help with stoma care was an important need for survivors with additional impairing comorbidities (e.g. poor vision, hernias, not seeing the stoma due to abdominal girth [34, 35] and (prolonged) weight loss [51]. For some, assistance with stoma care was not accessible due to finances or environmental restrictions (e.g. not living with a caregiver, inconvenient bathroom [35]). Generally, survivors with fewer caregiving needs reported less impact on being able to live full lives [34].

#### Supportive professional care and information needs

Need for supportive care from professional health services were reported in two papers [45, 50]. Survivors with a stoma ‘overwhelmingly discussed’ unmet needs related to their stoma including body image, sexual health, finding appropriate appliances, caring for the stoma, and costs associated with purchasing stoma supplies but many felt guilty contacting a healthcare professional about these issues and consequently did not seek professional help [50]. In one study, male rectal cancer survivors indicated interest in psycho-educational sexual health interventions [45]. In the studies of patients with a reversed stoma, supportive professional care needs were not discussed.

In general, survivors want tailored information and support with diet and physical activity from their healthcare providers [42]. The information that was provided was often inconsistent between healthcare professionals, poorly timed (e.g. need for information regarding impact of treatment on sexual functioning *prior* to treatment), did not cover all important topics (i.e. sexual functioning, exercise, nutrition, psychological well-being, follow-up care), or insufficient and difficult to understand (e.g. jargon, information overload) [36, 41, 42, 45, 50, 51].

#### Experience with health care

Only two studies explored CRC survivors’ experiences of healthcare [43, 50]. Survivors frequently felt guilty asking for professional help and thought they should deal with their problems themselves [50]. Consequently, they did not seek help for their unmet needs (e.g. sexual health, bowel function). When they did seek help, they experienced difficulty reaching stoma nurses/consultants, but viewed them as important for helping with stoma care, valuing their expertise. Survivors were comforted by continued specialist care specifically in terms of rapid access should they experience a recurrence, and felt that GPs should work more closely with other healthcare professionals, questioning whether GPs could order all appropriate tests and investigations for cancer follow-up. Some viewed GP-led care as an obstacle to quick access to cancer services [50]. Survivors ‘overwhelmingly’ described transition from active treatment to follow-up care as a shift in responsibility from the oncologist to the patient during follow-up care [42]. This caused feelings of distress and abandonment. In another study, survivors without a stoma felt unsupported to manage their bowel changes while survivors with a stoma received support from the oncology team/dieticians to manage bowel changes [42].

#### Lifestyle/health behaviour modifications

Lifestyle and health behaviour changes were reported in 11 papers [32, 33, 37, 39, 41, 43–46, 49, 51]. Survivors with a permanent stoma regulated the timing, amount and the kind of foods consumed to manage bowel functioning. For example, avoiding certain foods that caused gas and rapid evacuation [32], limiting food consumption for planned social events or traveling long distances, and taking immodium to only defecate at home [37]. Some survivors tried different stoma appliances to find ones that suited them best [33, 37]. Others used (daily) irrigation [37, 49], which was helpful for controlling the timing and location of defecation and enabled participation in social, sexual and occupational activities [37]. Some survivors with a stoma reported using anti-diarrhoeal to decrease “output”’, but others felt more comfortable modifying their diet than taking regular medication [51]. Other preventive measures to avoid potentially embarrassing stoma-related situations included packing extra supplies and wearing larger clothing to accommodate and hide the stoma [33]. Public bathrooms posed a particular challenge for stoma care due to toilets being too low, inability washing the stoma bag, cleanliness, and availability of toilet paper [33].

Survivors with reversed stomas developed strategies to manage their altered and often unpredictable bowel functioning. Strategies included diet changes such as refraining from certain foods or from (completely) eating [39, 41], increased fibre intake, structured eating times, having bowel movements at home, avoiding public restrooms, planning outdoor activities close to a bathroom, and developing a daily routine for bowel movements [45]. Others engaged in preventive measures such as use of anti-diarrhoeal, vitamins or fibre supplements or protective measures such as incontinence pads or carried a change of clothing in case of bowel accidents [39, 41, 45, 46, 49, 51]. Some reported beneficial effects while others were concerned about over-reliance on supplements, exacerbation of diarrhoea, and ‘sickly’ taste from the supplements [51]. Strategies were also found for skin care (e.g. use of barrier creams, moist toilet wipes) [39, 46]. CRC survivors at increased risk for cardiovascular disease did not feel the need to change their diet or physical activity to improve their health. They doubted the link between diet and cancer and had (mis)perception about benefits of physical activity, preferred to enjoy life (e.g. eating/food), perceived medical surveillance by their GP sufficient, lacked motivation or support to make changes, or had failed at attempts to change behaviour in the past [43, 44].

#### Financial toxicity

Four papers identified financial problems [32, 34, 35, 50], but no study specifically aimed to explore financial issues. The main financial concerns were inability to afford professional help or carer relief [32, 34]. Paid caregiving was ‘unaffordable or unacceptable’ for some families [35]. Survivors with stomas had costs associated with stoma supplies [50].

#### Employment/work experience

Six papers identified paid and unpaid work-related problems [32, 41, 44, 47, 49, 50], but no study specifically aimed to explore occupational issues. Survivors reported special needs and challenges to returning to work after CRC treatment [50]. Some changed careers due to inability to return to previous roles and responsibilities [44] or found ways to manage their symptoms to allow returning to work (e.g. changing diet or irrigation) [47, 49]. Others felt embarrassed and worried about possible ‘accidents’ at work [32]. Some chose not to inform their employer about their bowel symptoms for fear of being considered a burden or losing their job. These people tried to manage their symptoms discretely [41].

#### Proximal versus distal effects on health-related quality of life

Figure 4 illustrates how CRC and its treatments may affect a person treated for CRC in mid- and long-term survivorship. Proximal effects occur directly as a consequence of the CRC and/or treatment for the disease, such as gastrointestinal symptoms, pain and fatigue [52]. These may consequently affect a person’s ability to function and their overall sense of wellbeing, i.e. cause distal effects. Experiencing symptom burden can directly (i.e. proximal) impact psychological wellbeing, or indirectly, via experience of symptoms contributing to loss of functional ability.

## Discussion

### What it is like to be a survivor of CRC

We identified 20 papers reporting on 15 studies of survivors’ experience of mid- to long-term impacts of treatment for CRC. Evidence synthesised across studies indicated a range of bowel symptoms such as frequent and irregular bowel movements, loss of control over bowels, and faecal incontinence were the most common complaints that persisted long after treatment completion. Other common symptoms included pain, fatigue, disrupted sleep, and skin irritations. These persistent and often unpredictable symptoms have knock-on effects on peoples’ physical, role, social, emotional and sexual function. Work was considered an important part of social life, and many also had difficulty socialising, participating in everyday activities and returning to previous work duties. Particularly challenging was the need to be close to a toilet, with public bathroom facilities being suboptimal. These negative impacts can put a strain on relationships due to increased care-giver responsibility, changed roles, and altered sexual and intimate relationships. This in turn affected body image, sense of self and overall HRQOL. These are important findings as anecdotally, some clinicians who manage CRC patients believe most people treated for primary CRC fully recover from surgery by 12 months. Our review highlights that many CRC survivors experience persistent and long-term gastrointestinal symptoms and functioning impairments that often go unmanaged.

Perhaps surprisingly, symptoms and functional impairments were more problematic for people without a stoma or stoma reversal compared to those with a stoma. But having a stoma comes with its own challenges such as stoma care, out-of-pocket expenses for stoma supplies, embarrassment due to accidental leaking and smell, and concerns about disclosing the stoma and bowel symptoms to loved ones, family and colleagues. Having a stoma was perceived by some as a failure of treatment and something they were ashamed of. This finding is consistent with the broader survivorship literature where study participants found the experience of adapting to an ostomy daunting and the ‘lowest point in their cancer experience’ [53]. Others adapted well and reported the stoma allowed them to regain their functioning and improved their HRQOL once they found ways to accommodate and care for the stoma. Some wished that they had opted for an immediate stoma rather than delaying the procedure [53]. However, learning to live with a stoma could take years of trial and error with ‘accidents’ and loss of privacy and independence, contributing to feelings of embarrassment and helplessness [53]. What would be informative is group comparison, matching for age and gender, of long-term PROs between survivors with a stoma, those with a stoma reversal and those not needing a stoma.

Several papers reported that survivors used trial and error to find preventative and protective strategies to manage and adjust to their altered bowel functioning and stoma care, allowing them to participate in daily, social and work-related activities, and sexual relationships [33, 37, 39, 42, 51]. This finding is similar to breast cancer survivors who report adapting their clothing to avoid pain in the breast area following surgery [54]. Similarly, people with other chronic conditions report finding strategies to manage urinary incontinence such as modifying their fluid intake, changing underwear frequently, and using waterproof mattress protectors [55]. CRC survivors seem ill-prepared for how to manage long-term symptoms and treatment effects, and reports from survivors suggest these effects are underestimated by clinicians. It appears that many CRC survivors experience and manage their symptoms in isolation, without access to healthcare support and interventions. Finding the ‘right’ management strategy may be an individual process. Little is known about what health services and interventions are available and tried by CRC survivors to manage their symptoms and functions. Evidence is needed about health services and interventions that effectively control symptoms and improve function, to replace the long and often painful process of trial and error and to reduce unnecessary suffering.

### Limitations with current evidence

Our review provides an international perspective, representative of the views of 328 colorectal and rectal cancer survivors. However, our review had some limitations. Firstly, there may be informative individual studies that were excluded due to lack of reporting of time since diagnosis or treatment. We contacted authors in an attempt to obtain this information and addressed this limitation by comparing our review findings with key findings from the excluded papers. No notable differences were found. Secondly, our search was limited to full papers indexed in electronic databases. It is possible that relevant studies may have been retrieved through hand searching. Due to the large number of retrieved abstracts and limited details provided, conference abstracts were excluded. Despite some limitations, all studies included in our review were explicitly participants at least 12 months post-treatment completion (or 2 years since diagnosis), providing evidence about chronic and long-term treatment effects from the perspective of CRC survivors.

The evidence base also has some limitations. Generally, various aspects of study conduct were poorly reported, limiting the informativeness of included studies and resulting in the exclusion of a number of potentially relevant papers. For example, reporting of certain sample (e.g. mean age, gender, education, race) and clinical characteristics (e.g. treatment details, cancer stage, cancer site (% colon vs. % rectal)) was poor. Multiple studies included both colon and rectal cancer patients, but did not report their results separately (Fig. 1). Given that treatment varies for the two tumours, we cannot assume that their experiences and treatment impacts are equal. Further, many studies did not clearly report whether they asked participants about their current experience (i.e. at the time of interview) or to recall their past experience. Several papers did not report time since diagnosis or treatment completion and were consequently excluded because we were unable to confirm whether the study sample met our inclusion criteria (Fig. 1).

These analysis and reporting limitations precluded observations due to age, specific treatment for CRC, and disease type and stage, mainly because studies often pooled participants in the analysis or did not report findings by these variables. Further, no study considered the duration of certain problems that occurred after treatment completion or when they occurred relative to treatment end; rather, they pooled samples with a wide variation of time since end of treatment to data collection. Consequently, we do not know specifically which symptoms and functional impairments occur after treatment and persist long-term versus those that are not a problem after treatment completion but develop later on. Further, we cannot conclusively say anything about adjustment over-time and rates of recovery. Pooling PRO data collected at widely variable times since end of treatment completion (e.g. samples included patients anywhere from a few months post treatment up to 72 months post treatment), obscures patterns of acute impact versus long-term recovery. Pooling treatments received, participant ethnicity, and ages in the analysis precludes conclusions about differential treatment effects, possible differences in outcomes between minority groups, and any age-related differences, respectively. For example, because ages ranged from 44 - > 85, we are unable to differentiate urinary problems due to the disease and treatment from those that were a consequence of aging.

### Critical gaps in the evidence

A number of evidence gaps were identified. Only a few papers reported informal or healthcare supportive care needs. Those that did reported need for timely, relevant and tailored information about, and interventions to, manage GI symptoms and sexual function issues. Consistent with the broader CRC survivorship literature, survivors need advice and management plans for a range of physical and psychological effects such as bowel symptoms, nutrition and food intolerance, weight loss/gain, fatigue, sleep problems, sexual function, fear of recurrence and reduced mobility that are regularly reviewed and sensitive to bodily changes and ongoing treatment [56].

Only three studies [32, 36, 45] aimed to explore the impact of treatment for CRC on sexual function; two in permanent ostomy samples, one in a male-only rectal cancer sample, and none in homosexual men. No study specifically explored relationship dynamics and potential stresses placed on relationships from altered sex life following treatment for CRC. The main focus of studies has been on the personal impact of sexual problems on body image and feeling sexual and desired. Bowel symptoms, pain and having a stoma constrained intimacy and how couples supported (or not) each other. However, no study explored sexual functioning from the perspective of both survivors and their partners, or what supportive care both parties might need. A lot is known about sexual function issues in other cancers such as breast [57] and prostate [58] but contributors to sexual function problems and unmet needs are not well understood, documented and often unrecognised in CRC, and it appears that even less is done about it clinically.

We found no qualitative studies aimed at exploring experiences of people treated for CRC from minority groups, people diagnosed and treated for anal cancer, or young CRC survivors. Even though some studies included survivors aged under 50, results were not reported by age groups. Younger men may experience long-term effects that impact their HRQOL differently than older men, for example, in terms of fertility and family life, occupation, caregiving responsibilities, social functioning, and sexual activity. As reported in one study, older men (median age 67) identified sexual function as less of a concern [45].

Little is known about the financial toxicity and return to work needs as no studies specifically aimed to explore these issues in CRC survivors. Anecdotally, CRC survivors have many out-of-pocket expenses and specific needs related to returning to work. CRC survivors want to be informed about supportive care interventions that are not fully covered by public healthcare to enable them to better decide whether to proceed with options that require out-of-pocket expenses [59]. More work is needed in these areas to better support CRC survivors. Fatigue is a common problem in many cancers [60] including CRC but no study specifically explored this issue. Further, no study aimed to assess cognitive functioning, genetic or heredity issues. Finally, only three studies described caregiving demands, but these focused on women [36–38]. Based on partner support literature (women tend to take over caregiving role), we can assume that these results do not generalise to couples where the women is the patient and the man the caregiver. More research is needed.

### Conclusion

CRC survivors experience ongoing and often unpredictable GI symptoms and functioning impairments more than 1-year post-treatment completion. Better understanding of these chronic and late effects of treatment for CRC like bowel problems, distress and sexual issues is essential for overcoming existing shortcomings in cancer care of long-term CRC survivors. Survivors often self-manage their symptoms and functions rather than seek professional help. Self-management is not always successful and at times detrimental (e.g. exacerbate rather than alleviated symptoms). Consequently, many issues become chronic. What is unclear is why survivors choose to self-manage rather than seek help - is it that appropriate services and interventions do not exist or they do exist but survivors and health professionals do not know they exist or appropriate referral pathways are not in place. Providing adequate and timely information, setting realistic expectations about possible symptoms and impacts of treatments, and interventions to manage GI symptoms and sexual function issues may reduce some negative psychological effects. Importantly, instead of the long and often painful process of trial and error, evidence about which health services and interventions are effective in treating symptoms and improving functions is needed to reduce unnecessary suffering. Follow-up care for CRC survivors should integrate screening for likely long-term effects and provide targeted supportive care.

## Data Availability

*Not applicable.*

## Abbreviations

COREQ: Consolidated criteria for reporting qualitative research
CRC: Colorectal cancer
IOM: United States Institute of Medicine
HRQOL: Health-related quality of life
PRO: Patient-reported outcome

## Footnotes

1 XX, YY, ZZ used to replace author initials for blind manuscript
